# Understanding caretakers' dilemma in deciding whether or not to adhere with referral advice after pre-referral treatment with rectal artesunate

**DOI:** 10.1186/1475-2875-9-123

**Published:** 2010-05-12

**Authors:** Daudi O Simba, Deodatus C Kakoko, Marian Warsame, Zul Premji, Melba F Gomes, Goran Tomson, Eva Johansson

**Affiliations:** 1 Department of Community Health, Muhimbili University of Health and Allied Sciences, Dar es Salaam, Tanzania; 2 Division of Global Health [IHCAR], Department of Public Health Sciences, Karolinska Institutet, Stockholm, Sweden; 3 Department of Behavioural Sciences, Muhimbili University of Health and Allied Sciences, Dar es Salaam, Tanzania; 4 Department of Medical Parasitology and Entomology, Muhimbili University of Health and Allied Sciences, Dar es Salaam, Tanzania; 5 UNICEF/UNDP/World Bank/WHO Special Programme for Research and Training in Tropical Diseases [TDR] Geneva, Switzerland; 6 Medical Management Centre [MMC], Karolinska Institutet, Stockholm, Sweden; 7 Nordic School of Public Health, Gothenberg, Sweden

## Abstract

**Background:**

Malaria kills. A single rectal dose of artesunate before referral can reduce mortality and prevent permanent disability. However, the success of this intervention depends on caretakers' adherence to referral advice for follow-up care. This paper explores the dilemma facing caretakers when they are in the process of deciding whether or not to transit their child to a health facility after pre-referral treatment with rectal artesunate.

**Methods:**

Four focus group discussions were held in each of three purposively selected villages in Mtwara rural district of Tanzania. Data were analysed manually using latent qualitative content analysis.

**Results:**

The theme "Caretakers dilemma in deciding whether or not to adhere with referral advice after pre-referral treatment with rectal artesunate" depicts the challenge they face. Caretakers' understanding of the rationale for going to hospital after treatment - when and why they should adhere - influenced adherence. Caretakers, whose children did not improve, usually adhered to referral advice. If a child had noticeably improved with pre-referral treatment however, caretakers weighed whether they should proceed to the facility, balancing the child's improved condition against other competing priorities, difficulties in reaching the health facilities, and the perceived quality of care at the health facility. Some misinterpretation were found regarding the urgency and rationale for adherence among some caretakers of children who improved which were attributed to be possibly due to their prior understanding.

**Conclusion:**

Some caretakers did not adhere when their children improved and some who adhered did so without understanding why they should proceed to the facility. Successful implementation of the rectal artesunate strategy depends upon effective communication regarding referral to clinic.

## Background

Malaria, a preventable and curable disease, still accounts for an estimated 250 million clinical disease episodes with nearly one million deaths of which about 90% occur in Africa in children under five [1]. In Tanzania, malaria is a leading cause of morbidity and mortality accounting for 30% of hospital admissions and 15% of hospital deaths [Ministry of Health and Social Welfare, Tanzania. Annual Health Statistical Abstract. 2006].

Many children with severe malaria do not reach a hospital in time due to limited geographical access to health facilities [2,3]. Since acute malaria can progress rapidly in young children leading to a high case-fatality rate, especially among those living far away from hospitals, rapid treatment is important to avert death [4]. High mortality in malaria also results from reluctance of caretakers to take children with convulsions to hospital for fear of injections [5]. When children with severe malaria cannot be treated orally and referral is likely to take some hours, a single dose of rectal artesunate before referral reduces the risk of mortality and permanent disability [6]. Adherence to advice to proceed to the nearest facility for further assessment and management of the patient is critical to achieving the benefit of rectal artesunate pre-referral treatment. Follow up treatment is necessary to complete the treatment of malaria infections and to reduce the development of resistance [7], while diagnosis at the facility is necessary to confirm the infection and manage non-malaria conditions [6].

A recent multi-centre trial on rectal artesunate given as a pre-referral treatment to patients with suspected malaria who could not take oral drugs at the community level reported high adherence; however, between 5-12% in Ghana and Tanzania who survived for six hours never went to a clinic [6]. Severity of symptoms, costs, use of referral cards and health workers communication skills have been shown to influence adherence to referral advice when patients presenting at primary health care facilities are advised to proceed to a secondary facility [8,9]. There is very little information on compliance with advice to proceed to a facility when such advice is given at the community level [6]; in the only community study where factors influencing compliance were assessed, severity of symptoms, and ability to recall advice given were two main factors increasing compliance with adherence advice [10]. The factors that provoke *non-adherence *still remain an open question.

This paper describes the results of a qualitative study undertaken to improve understanding of caretakers decision making on whether or not to adhere to the referral advice after their child had been given pre-referral treatment with rectal artesunate. Such information can be used to inform health education and policy with regard to compliance.

## Methods

### Study design and settings

The study was conducted in August 2007 in Mtwara Rural district, Mtwara region. The profile of the study area including location, administrative structure, socio-economic profile have been described elsewhere [10]. The Makonde tribe is the main ethnic group, other small groups include the Yao and the Makua. Kiswahili and Kimakonde are the main languages spoken by the inhabitants. Transmission of malaria in the district occurs throughout the year, the main species is *Plasmodium falciparum*. A majority of the people situated on the mainland are subsistence farmers living at subsistence level through small-scale farming, growing cassava as their main food crop and cashew nuts as a cash crop. Those living along the coast of the Indian Ocean are mainly small-scale fishermen.

There is no district hospital and patients are referred directly from the dispensary and health centre level to the regional hospital (Ligula Hospital). In the community, there are two trained Village Health Workers (VHW) in each village (a male and a female) selected by the village government and trained for three months on prevention of common diseases and treatment of minor illnesses. Although the VHW's role is mainly preventive, they often are engaged by national programmes and projects in curative services on an ad hoc basis. There are also traditional healers in the district, who also treat symptoms suggestive of severe malaria using various methods such as herbs and divine healing. Although, there are no strict regulations for registration in the country, estimates from the Ministry of Health and Social Welfare, Tanzania, indicate that there are more traditional healers in the country than clinicians.

### Study design

The study was conducted after a quantitative study [10]. Both the qualitative and quantitative studies were nested within a controlled deployment study of rectal artesunate that sought to determine the feasibility of VHWs and young mothers to provide rectal artesunate to febrile children with danger signs who could not take drugs by mouth. The quantitative data indicated that about one-quarter of the caretakers (primarily caretakers of children who did not have symptoms of altered consciousness or convulsions) did not comply with instructions to proceed to the nearest facility after pre-referral treatment with rectal artesunate. Qualitative methods were chosen to obtain an in-depth understanding of how various factors influencing adherence to referral advice operate.

### Participants

Three villages in the controlled deployment study, where rectal artesunate was made available were purposively selected for focus group discussions (FGDs) with parents of young children; one village along the coast (Village 1) and two from the inland one from the northern side (Village 2) and one from the southern part (Village 3) of the district. Caretakers of under fives, with and without experience of pre-referral treatment were included. In each village separate discussions were held with groups of (i) female caretakers, whose children had been treated, (ii) female caretakers, whose children had not been treated, (iii) male caretakers, whose children had been treated, and (iv) male caretakers, whose children had not been treated with an emergency dose of artesunate suppository medicine, giving a total of 12 FGDs in the three villages. The CDDs were able to identify caretakers, whose children had or had not received pre-referral rectal artesunate. From the families of treated children, both male and female caretakers were invited except, in a few instances where the family had only one caretakers; in these cases a male caretaker was invited to the male treated group and a female partner was invited to the female treated group. For the non-treated families, only one member of the family was invited; this was purposively done to increase variability. While parents with experience of the drug were targeted to provide information leading to adherence or non-adherence to referral advice, parents of children who were not treated were included to triangulate information. The caretakers of non-treated children were considered to bear witness to events occurring when their relatives or neighbours had a child with symptoms requiring pre-referral treatment. In rural communities where people interact closely, people are well informed, and often involved in the care of a sick neighbour or relative [11] and, therefore, can serve as good informants.

Participants were invited on the day when the FGD was held. The community treatment providers, involved in the intervention study (CDDs), and village leaders were asked to invite people of mixed characteristics (different religion, daily preoccupations, locality and household characteristics) to these meetings. While support from the CDDs was necessary because they knew families of treated children, support from village leaders was crucial in gaining entry to the community. Individual informed consents were sought and obtained from the participants just before the discussions started. This was done after the CDDs and village leaders had gone in order to increase caretakers' freedom of choice to participate. Those who did not know how to read or write had the text read by the moderator and provided fingerprinted consent. Only one participant needed more information on the rationale for signing the form. Between 6 and 10 participants were invited to each group. The age range was 22-73 [mean 37.0] for female caretakers and 21 - 75 [mean 33.9] for male caretakers, which was lower than the moderator who was 50 years old. The age of the moderator and the wide age range of the participants did not deter the younger participants from communicating freely in the FGDs, because the issue of treatment of malaria is not a taboo in the settings hence the topic could be communicated among people of different age groups. In fact, the varied age range in these FGDs helped to create dynamic discussions. In total, 84 participants were involved, of which 44 were females and 40 were males as shown in Table 1. All the participants were peasants engaged in small-scale farming and nearly half of them had primary level education, while the rest had not attended a formal school.

**Table 1:** Participants' characteristics by village [n = 84]

	Total number	Number and % of caretakers of treated children	Number and % of women	Number and % of caretakers with primary education
Village 1	34	17 (50.0)	19 (55.9)	14 (41.2)
Village 2	26	14 (53.8)	13 (50.0)	11 (42,3)
Village 3	24	12 (50.0)	12 (50.0)	12 (50.0)
Total	84	43 (51.2)	44 (52.4)	37 (44.0)

### Procedures

Focus group discussions (FGDs) were used for data collection because of the advantage of opening up discussions without personalizing opinions as well as obtaining a community perspective on the issues discussed [12]. The study was carried out after conducting a quantitative study [10] to allow for preliminary analyses and to focus the interviews. The questions were pre-tested in Mbuo village and results were used to modify the interview questions. Discussions were held in a quiet place in the villages, conveniently selected by village leaders. Participants were introduced to the objective of the FGDs and the FGDs ranged from one to two hours [12]. Discussions normally ended when no more new information was elicited. Discussions were moderated by one of the investigators (DK), using an interview guide with pre-tested questions based on the categories obtained from the preliminary analysis of the quantitative data.

The discussions focused on participants' perception of reasons for and against seeking further treatment after the pre-referral dose of rectal artesunate. Detailed information was sought, especially where opinions, ideas or meanings varied. Similar but modified questions were asked of caretakers whose children had not been treated: e.g. 'why do some people not seek further treatment at a health facility?' The discussion guide was prepared in English and translated into Kiswahili by DS and DK. Discussions were held in Kiswahili, a language spoken by most people in the villages. Sometimes, local terms in 'kimakonde' language were used when the participants could not get the best Kiswahili words to make themselves better understood. In these cases, the RAs proved to be useful in translating these terms to the moderator. The moderator (DK) was assisted by two research assistants, one taking notes and another making observations. DS made observations during the discussions, which were conducted in Kiswahili and were tape-recorded. One FGD was executed each day to be able to reflect and consolidate emerging issues for further questioning. A decision not to include further villages was taken when information had reached saturation, i.e. no new information was obtained [13].

### Data analysis

Tape recorded discussions and discussion notes were transcribed, translated into English and thereafter back into Swahili by two translators. DK and DS supervised transcriptions from the tapes and the translations into English. Latent qualitative content analysis was carried out following the guidelines by Graneheim and Lundman [14] without the use of electronic software, because of its ability to consider variations in opinions among participants. DS initiated the coding and category assignments and four other co-authors went through them identifying areas of discrepancies. Differences and discrepancies were discussed and consensus reached after referring back to the tapes. Coding and categorization was initially done for the whole text and later sorted by gender and whether the caretakers had a treated child or not.

### Trustworthiness

#### Reliability of information

A good rapport with village leaders and members of the community had been created during the intervention study. Female participants were separated from male caretakers so as to give opportunities to each group to express themselves freely. Note takers were indigenous persons and helped to decipher the local vernacular and jargon, which facilitated translation of information. The involvement of an experienced social scientist (DK) moderating the FGD ensured that most participants were given the opportunity to speak.

#### Consistency

was ensured by the use of the same moderator and note takers in all FGDs. DS and DK prepared the condensed meaning units and codes and discussed areas of significant deviations. Other co-authors were provided with the scripts and gave feedback on the text and codes and they were also involved in identification of the theme, categories and sub-categories.

#### Reflexivity and triangulation

Researchers influence was minimized by having a mixed team: malariologists (ZP and MW); social scientist experienced in health promotion and behaviour (DK); public health researchers (DS and EJ) and a health systems researcher with international and local experience (GT). The wide professional variations and geographical background of the researchers also helped to triangulate information during analysis, giving both the insiders' and outsiders' perspectives. Involvement of caretakers with treated versus non-treated children enabled triangulation of information since the caretakers of the non-treated children were independent observers of what transpired at the time of illness.

#### Feedback

Only one FGD was conducted per day, normally in the morning. Contentious issues arising from the discussions were discussed in the afternoon and introduced as probing questions for verification during the next day and subsequent FGDs. Preliminary findings were presented at the Health Systems and Policy research group at the Karolinska Institutet and feedback obtained was used during analysis and in drawing up relevant themes.

### Ethical issues

Ethical approval for the study was obtained from the Muhimbili University of Health and Allied Science and from the National Institute of Medical Research. Permission to conduct the study was obtained from the regional, district and village authorities. Individual informed consent was sought and obtained from the participants prior to FGDs. Those who did not know how to read or write had the text read by the moderator and provided fingerprinted consent. A few needed further explanation before consent.

## Results

Two categories and four subcategories were developed (Table 2). No major differences were noted between the different groups, treated versus non-treated, male versus female. Results from the FGDs are discussed and supported by quotations to illuminate some of the findings from each subcategory that highlight the experiences and perceptions influencing the decision to adhere (or not) to referral advice. "Caretakers' dilemma in deciding on whether or not to adhere to referral advice after pre-referral treatment with rectal artesunate" summarizes the thought processes that caretakers experienced when making the decision to go or not to go to a clinic after pre-referral treatment. This formed the theme of the findings. The caretakers' understanding of referral advice as well as perceptions of the child's condition weighed against competing priorities; difficulties in reaching the health facility and perceived quality of care appeared to be key to this decision. These two judgements are provided in two main categories, subdivided into four subcategories seen in Table 2.

**Table 2:** Summary table of results

THEME	Caretakers' dilemma in deciding on whether or not to adhere to referral advice after pre-referral treatment with rectal artesunate			
CATEGORIES	Understanding the referral advice		Weighing the child's condition against other competing priorities; difficulties in reaching the health facility and perceived quality of care	
SUBCATEGORIES	When? [Understanding the urgency of the referral]	Why? [Understanding the reasons for the referral]	Weighing child's condition against competing priorities and difficulties in reaching health facility	Judging the quality of care at health facility
CODES 1	Go if condition changes	Go to facility for further investigation and treatment	Distance to facility is a deterrent if child improves	Referrals get first priority
2	Go as soon as possible	Go to facility for fear of symptoms recurrence	Priority can be given to other activities if child improves	Doctors do not care
3	Rest before going	Not go to facility because child condition has improved	Transport not a deterrent if child does not respond	Informal payment

### Caretakers' dilemma in deciding on whether or not to adhere to referral advice after pre-referral treatment with rectal artesunate

Factors that can influence caretakers to adhere to referral advice can be either favourable or unfavourable. The FGDs revealed four types of actions that caretakers took depending on child's condition and reason for the decision. These were: (i) Caretakers, whose children's condition improved after pre-referral treatment, who consequently saw no reason to proceed with the referral advice given; (ii) Caretakers, whose children's condition improved, but who adhered with referral advice without a clear understanding of the rationale behind this advice; (iii) Caretakers, whose children's condition improved, and who proceeded to the clinic with the aim of seeking further treatment as advised; and (iv) Caretakers, whose children did not improve and adhered to referral advice.

The main theme is described below using the categories and subcategories as follows:

### Understanding of referral advice

Caretakers' understanding of why and when to go was important for prompt adherence.

### When? Understanding the urgency of the referral

Understanding of when to go to the facility varied. Some participants reported having been advised to go as soon as possible or on the same day; others reported being advised to rest before taking the child to the health facility, as illustrated in the three quotations below.

*"The information they gave was for the parents to take the kid to the dispensary as soon as possible"*. [Male caretaker of a child treated with rectal artesunate]

*"... I was told 'if the fever becomes very severe you should take her to the hospital, if the condition is somehow fine you have to rest a bit and take her later to the hospital''*. [Female caretaker of a child treated with rectal artesunate]

*"After the child was attended, the health worker [CDD] told me to monitor the child's condition at home; and if it got worse, I should send her to the dispensary, otherwise I could stay home. And the child got better, so I did not take her to the dispensary"*. [Male caretaker of a child treated with rectal artesunate]

Some participants argued that their lack of understanding about when to go to the health facility might have been due to the fact that not all members of the village had had a chance to attend the community sensitization gatherings and among those who did, some had a low level of comprehension, which might have compromised their understanding of the messages. As one of the respondents stated:

*"... I feel that more sensitization should be done using leaflets and meetings since the community here is very large. If a sensitization programme takes one day, it is very possible that some people are not around on that day, or sometimes some people have less ability to understand, so the message must be repeated again and again to be properly understood". *[Male caretaker of a child treated with rectal artesunate]

### Why?: Understanding the reasons for the referral

The CDDs were trained to explain to caretakers the reasons for the referral, which included investigations to establish diagnosis and need for the complete consolidation treatment regime if the diagnosis was malaria, as the child could recrudesce. It was important that caretakers understood the rationale for adherence and the possible consequences of non-adherence. They were asked to seek care from a facility because the child could have a different disease or have another disease in addition to malaria. Therefore some went for fear that the disease might recur.

*"I went for laboratory investigations so as to determine the number of malaria parasites affecting the child. It is possible that a child might get better briefly and after two days the condition may recur. That is why they advise that you should go to the dispensary even when you feel that the child is getting better"*. [Male caretaker of a child treated with rectal artesunate]

Some participants reported going to the health facility because of the advice given by the CDDs in whom they trusted because of the knowledge they had received from experts.

*"I took my child to the dispensary as advised by the service provider [CDD]... I did not go there [to the health facility] just because the condition did not improve, no, it was because I was directed to do so by the service provider"*. [Male participant of a child treated with rectal artesunate]

Some of the participants did not adhere despite acknowledging having received referral advice from the CDDs.

*"... mine had signs in the night and was given suppository insertion drugs and I was told to take the child to the dispensary next morning. But next morning I found the child's condition was better, so I did not go... I trusted the drug"*. [Male participant of a child treated with rectal artesunate]

In the course of the focus group discussions several misunderstandings were unveiled regarding the need for the follow up investigations and treatment. According to guidelines all children were asked to be taken to the health facility after insertion of rectal artesunate irrespective of their condition. However, some of the FGD participants thought that only a child whose condition did not improve, was supposed to be taken for further investigations. Others thought that through investigations they would be able to tell whether the child had malaria or convulsions, i.e. it was not appreciated that convulsions were a manifestation of severe malaria. There were also misunderstandings about whether a single dose of rectal artesunate was adequate to treat malaria/convulsions. While some participants perceived that a single artesunate dose was not adequate and was only meant to give some relief before reaching the health facility, others thought the treatment dose was sufficient.

*"I did not understand why I should take the child to the health facility when he had already been attended; I wondered what else was I going to do there?" *[Male participant of a child treated with rectal artesunate]

Participants, who perceived that the dose was not sufficient, reported having received the information from either the CDDs or through the sensitization meetings held in the villages prior to the intervention study. Those, who perceived the dose as adequate and did not follow referral advice, were mostly influenced by the improvement of the child's condition.

*"Personally, after the child was attended, the health attendant [CDD] told me to observe the child's condition at home and that, if it got worse I should send him/her to the dispensary, otherwise I could stay home. The child got better so I did not take her to the dispensary" *[Male participant of a child treated with rectal artesunate]

Further misinterpretations arose on the rationale for the referral note given to each parent designed to facilitate communication of information between the CDD at the village and the health workers at the health facilities. There were participants, who adhered to the referral advice because they perceived taking the referral card to the facility was the primary reason for the referral to a clinic.

*"... the form that she [CDD] gives means that you must take it to the dispensary... even if the child will not be taken there but it is important to take that paper to the hospital. Because you never know... the condition might recur and when you go back you will be told to go straight to the dispensary." *[Male participant of a child treated with rectal artesunate].

### Weighing the child's condition against other competing priorities; difficulties in reaching the health facility and perceived quality of care

Beneath the background of understanding and misunderstanding the urgency and rationale for adhering to referral advice, caretakers were faced with the challenge of assessing the child's condition against obstacles in reaching the health facility and the perceived quality of care at the health facility.

### Weighing the child's condition against competing priorities and difficulties in reaching the health facility

Most participants spoke in favour of rectal artesunate. They included those whose children were not taken for rectal artesunate but who had experience from their relatives or neighbours' children. Reporting the dramatic improvement of a nephew's condition after the insertion of rectal artesunate, one of the participants said:

*"...He [the child] was very weak and he was unconscious... When you lifted him up his hands became like......he didn't have power... When you called him he didn't respond... if you positioned his neck at any angle it just remain there... they took him to the service provider [CDD]... they inserted him the medicine. It took half an hour... he regained his power and was able to stand up and sit down." *[Female participant of a child not treated with rectal artesunate]

It was therefore not surprising that some caretakers cited improvement of a child's condition as the reason for not adhering to referral advice. For some of the caretakers, the perception was that if the symptoms did not continue it meant that treatment had worked and the child had recovered and it would be just a waste of time to go to the health facility.

*"For me I didn't go to the hospital. After they inserted the medicine, I saw my child's condition was okay, so I changed my mind". *[Female caretaker of a child treated with rectal artesunate]

Most of the caretakers of the treated children acknowledged being advised by CDDs to go to the health facility after insertion of rectal artesunate, but said they changed their minds later after seeing that the child's condition had improved.

*"She [CDD] told me to take the child to the hospital, but the child's health condition was good so I didn't make any further efforts *[Female caretaker of a child treated with rectal artesunate]."

In this situation where the child's condition had shown a remarkable/dramatic improvement other competing priorities took precedence. Some caretakers perceived adherence to referral advice as an impediment in their struggle for daily income. To them, the child's improvement became a double blessing - saving money and time - as some of the participants said:

*"But if I have something else to do so I find that it is better to engage in another activity... because we have got the services and the condition has improved then let me embark on other activities." *[Male participant of a child treated with rectal artesunate]

This study was conducted in villages that were situated more than 5 km from a nearby health facility, thus, some caretakers reported walking 10 km to reach a health facility. Although distance was reported by some participants as a deterrent to adherence to referral advice, especially if the child condition was not bad, others thought it was not a deterrent if the child was sick:

*"Going to the health facility does not-need transport cost. One can walk even to town [30 km] if she/he has a sick child"*. [Male participant of a child not treated with rectal artesunate]

### Judging the quality of care at health facility

The way health workers interact with patients, availability of drugs and use of the referral note were some of the issues that, although not reported explicitly, could have influenced caretaker's decision to adhere to the referral advice or not. There were participants who reported receiving good care despite the few numbers of clinicians. One of the facilities that had only one clinician was reported to cater for about five villages each with an approximate population of 10,000. Participants reported getting first priority when they reported with a referral note at the health facility as one of them describes:

*"If two of you enter the doctor's office you show the paper [referral note] and the one with no paper will be asked to leave and they give you the services quickly"*. [Male participant of a child treated with rectal artesunate].

There were also participants, who reported receiving unsatisfactory services as one of the participants reported '*... they wear gloomy faces and are not interacting, making us feel uneasy'*. Other participants reported being turned back to CDDs at the village for services thus defeating the purpose of the intervention.

*"I went to the dispensary before suppository treatment, and at the dispensary they asked me if the child had received suppository treatment... I said no. So they told me to go back to the service provider [CDD] for suppository treatment"*. [Male participant of a child treated with rectal artesunate]

Participants reported getting drugs at the health facilities, although constant shortages were reported and caretakers were asked to go and buy drugs from drug shops. Sometimes they were given a starting dose and asked to go and buy the remainder of the course. One of the participants reported having initially sought care from a health facility and was given a starting dose of 'panadol'. She then decided to go to the CDD in the village and her child was treated with an artesunate suppository.

Some of the participants did not go to the health facility, when they felt the child had improved fearing costs as well as concern that going to the health facility could consume time from productive chores. In relation to this, one of the participants said:

*"Because of difficulties in getting daily income, if the child improves... we think suppository medicine is adequate"*. [Male participant of child not treated with rectal artesunate]

Although many participants reported having received services free of charge, which is in accordance with the government policy, some of them seemed unaware of the policy and reported having paid user fees for children under the age of five. Thus many of the caretakers reported that they paid Tsh 100 (about 10 US cents) for syringes. They said that sometimes they did not have enough money and, therefore, some of them had to go without medicine or opt for herbs.

*"... there are people who are very poor and cannot afford the 100 shillings... some children might need five injections so you need five syringes... So you may need five hundred shillings, and this is not easy"*. [Male of participant of a child not treated with rectal artesunate]

Some participants reported that health facility workers ask for money [nicknamed as money for 'eggs', beans', and 'Fanta'] before treating the patients. One of the participants stated *"When my child gets sick, to him it is body fever but to me it becomes 'pocket fever" *[Male participant of a child not treated with rectal artesunate]. Ironically, some caretakers expressed satisfaction with this 'arrangement' arguing that it assures them of getting better services:

*"When he [the doctor] asks to be given two hundred [shillings], I think it is good because the child will get treatment... we don't know the procedure so if the doctor delays your treatment you think it is purposely"*. [Male participant of a child not treated with rectal artesunate]

## Discussion

This study suggests that the condition of the child following pre-referral treatment with rectal artesunate influences a caretakers' decision to adhere or not to referral advice. Among the caretakers of children whose condition improved, there were those who adhered and fully understood the advice given, caretakers who adhered without having a clear understanding of the reasons behind the recommendation and *caretakers who did not adhere mainly because the child's condition had improved*. A quantitative study, conducted prior to this qualitative study, showed that severity of symptoms *prior *to rectal artesunate treatment was the most important factor influencing adherence to referral advice [10]. A significant finding from this qualitative study is the fact that the condition of the child *after *rectal artesunate pre-referral treatment modified caretaker's decision on whether or not to adhere to referral advice.

In many discussions, participants who did not adhere to referral advice stated that they were uncertain about whether they were required to go to the health facility once the child's condition had improved, while others thought the drug was sufficient for cure. Uncertainty about whether all patients were required to be taken to the clinic was dominant among those who did not follow referral advice. This does not indicate that there was a lack of clarity in the messages given during the CDD-caretakers' interaction (although what transpired between the CDDs and caretakers is not known and it has been observed that caretakers can misrepresent or be reluctant to admit misunderstanding what health workers say [15]); whatever the reason, there appeared to be sufficient uncertainty in the provider-patient communication to tip the balance towards non-compliance with referral once the child got better [15,16]. Thus, the lack of consensus (by those whose children improved) regarding the urgency and the rationale for adhering to referral advice might also be a real consequence of their inadequate comprehension.

Some caretakers perceived that a single dose of rectal artesunate was adequate to cure malaria/convulsions when it was actually provided as a single dose before further management after reaching the facility. Others reported that they were told to go to the health facility if the child's condition did not improve, when in fact they were supposed to go regardless of the child's condition. It has been argued, that the interpretation of a message may be based on knowledge of a person's background and thinking framework [17,18] and that even if the CDDs provided information to caretakers that they were trained to provide, the caretakers' interpretation of the information might be different from the original message because they would process the new information based on their previous understanding of the subject [18]. Thus, the lack of consensus among FGD participants on when and why to adhere to referral advice might be due to variances in caretakers' prior knowledge.

Competing priorities such as house chores, distance to the health facilities, informal payments and inadequate quality of care at the health facilities were mentioned by some of the participants as reasons for failure to adhere to referral advice. Other research has reported affordability of care, availability of drugs, geographical accessibility/travel time, appropriate opening hours as factors influencing adherence [8]. Education, age, sex, quality of care [9] and charging for services also influence adherence [10].

The finding that caretakers whose children's condition did not improve after rectal artesunate pre-referral treatment, and that some of those whose children's condition improved, adhered to referral advice is encouraging in the implementation of the rectal artesunate strategy. However, the finding that some caretakers did *not *adhere to referral advice because their child had improved, poses a threat to the successful implementation of the strategy. Studies have shown, that the majority [90%] of children have a drop in temperature and over 90% parasite reduction within 24 hours after an initial dose of an artemisinin-based drug [19,20], but only about a third of the children will have cleared parasites from the blood [21]. Failure to take curative treatment in recommended doses after pre-referral artesunate would encourage recrudescence [19] and although resistance to a single dose of artemisinin is unlikely the high parasitaemia common among children with severe malaria could provide selective pressure for the emergence of resistance [22]. These outcomes will eventually undermine drug efficacy and caretakers' perception of the drug's efficacy [23,24]. The finding that caretakers whose children's condition improved adhered 'just for the sake of it', could also pose a threat to implementation of the strategy because failure to appreciate the rationale for the referral could be followed by defaulting with referral advice in the course of implementation.

Since referral alone is not enough to save life unless the patient reaches medical care very quickly [6] a good communication strategy is needed to encourage adherence [25,26]. The additional risk of recrudescence and drug resistance and the limitations of pre-referral rectal artesunate in curing malaria should be communicated.

## Conclusion

Some caretakers did not adhere when their children improved and some who adhered did so without understanding why they should proceed to the facility. Successful implementation of the rectal artesunate strategy depends upon effective communication regarding referral to clinic.

## Competing interests

The authors declare that they have no competing interests.

## Authors' contributions

DS, DK, MW, ZP, GT and EJ took part in designing the study, tools development, data analysis and manuscript writing. DS and DK were also involved in the field work. All authors approved the final manuscript.
